# Butyrolactone-I from Coral-Derived Fungus *Aspergillus terreus* Attenuates Neuro-Inflammatory Response via Suppression of NF-κB Pathway in BV-2 Cells

**DOI:** 10.3390/md16060202

**Published:** 2018-06-07

**Authors:** Yuan Yuan Zhang, Yi Zhang, Yuan-Bei Yao, Xiao-Ling Lei, Zhong-Ji Qian

**Affiliations:** 1 College of Food Science and Technology, Guangdong Ocean University, Zhanjiang 524088, China; zyyla92@126.com (Y.Y.Z.); hubeizhangyi@163.com (Y.Z.); 13286999640@163.com (Y.-B.Y.); 2 Shenzhen Institute of Guangdong Ocean University, Shenzhen 518108, China; 3 College of Chemistry and Environment, Guangdong Ocean University, Zhanjiang 524088, China

**Keywords:** butyrolactone-I, *Aspergillus terreus*, microglia, neuro-inflammation

## Abstract

Butyrolactone-I (ZB5-1) from the coral-derived fungus *Aspergillus terreus* was investigated in this study to estimate its anti-neuroinflammatory effects on lipopolysaccharide (LPS)-induced BV-2 microglia cells. MTT assay indicated that ZB5-1 in tested concentrations had no cytotoxicity on BV-2 cells, and significantly reduced the production of nitric oxide (NO), measured using Griess reagent, and interleukin-1 beta (IL-1β), detected by enzyme-linked immunosorbent assay (ELISA). ZB5-1 also down-regulated the expression of inducible nitric oxide synthase (iNOS) and cyclooxygenase-2 (COX-2) in a dose-dependent manner by Western blot analysis. Moreover, the effect of ZB5-1 on the nuclear factor-κB (NF-κB) signaling pathway was studied via the expression of phosphorylation of NF-κB p65 and inhibitor of NF-κB (IκB), and the nuclear translocation of NF-κB p65 respectively. The results showed that ZB5-1 could inhibit the phosphorylation of p65 and IκB. Furthermore, molecular docking study suggested that ZB5-1 bound at the active sites of NF-κB to prevent its translocation to the nucleus. Therefore, we suggest ZB5-1 has a potential to reduce the anti-inflammatory response in LPS-induced BV-2 cells.

## 1. Introduction

Microglia cells are ubiquitously distributed in the central nervous system (CNS) [1], functioning as the brain’s resident macrophages to maintain brain homeostasis and protect the brain from infections and stimuli [2]. However, over-activation of microglia cells can release overproduction of pro-inflammatory cytokines, reactive oxygen species (ROS), and nitric oxide (NO), which can cause neuro-inflammation, a chief culprit of neurodegenerative diseases [3]. Therefore, for the sake of the development of therapeutic agents for neurodegenerative diseases, the exploration and identification of compounds that can suppress microglia cell activation has become a key process [4].

Up to now, it is estimated that nearly 100,000 fungal species have been discovered. Due to the species’ diversity and the innovation of chemical, biological, and genetic approaches, fungi still represent the richest source of new metabolites. Additionally, an increasing number of endophytic and marine-derived fungi have been studied in recent years [5]. Such fungi provide a silver lining for new natural products screening, however, many potentials still need to be discovered [6]. The filamentous ascomycete fungus *Aspergillus terreus* is widely present in both marine and terrestrial creatures [7]. It is famous for its two valuable metabolites: Lovastatin and itaconic acid. The former is a cholesterol-lowering drug, while the latter is widely used in polymer manufacturing [8]. In addition, many researches have been conducted to explore and identify more lead compounds derived from *A. terreus*. In terms of structural or biogenetic types, those compounds are classified into polysaccharide [9], peptide [10,11], terrain [12,13], butyrolactone [12,14], meroterpenoids [15,16], and other compounds with novel and complex structures.

Butyrolactone-I (ZB5-1) was obtained from *A. terreus* XWC21-10, an endophytic fungus isolated from Puko Shore coral (*Porites pukoensis*) in Xuwen (Guangdong, China). As a scleractinian coral, *Porites* account for 57% of total species in the Xuwen coral conservation area. At present, a few researches focus on *P. pukoensis*. One study isolated 23 fungal strains from *P. pukoensis*. These strains belonged to 10 genera, of which *Aspergillus* sp. occupied 30.4%. In addition, the fermentation supernatant and mycelia of these fungi presented antibacterial activities [17]. Previous studies discovered and tested the anti-inflammatory effects of *A. terreus* derived compounds, such as butenolide derivatives [18,19] and meroterpenoids [20]. A butyrolactone I analogue, asperteretal A, significantly decreased the NO production in RAW264.7 cells [19]. Recently, Liu et al. [18] reported that butyrolactone I exhibited the inhibitory effect on NO, which is close to indomethacin.

Based on these results, in order to determine the molecular mechanism underlying the anti-inflammatory of ZB5-1, the present study estimated the anti-neuroinflammatory effects of ZB5-1 in BV-2 cells by in vitro experiment and molecular docking.

## 2. Results

### 2.1. Identification of ZB5-1

Butyrolactone-I was obtained as a yellowish oil by repeated column chromatography of the fermentation extract of marine fungal strain *A. terreus* XWC21-10. Its NMR and specific optical rotation data are listed as following and compared with a previous report [21].

**Figure 1:**
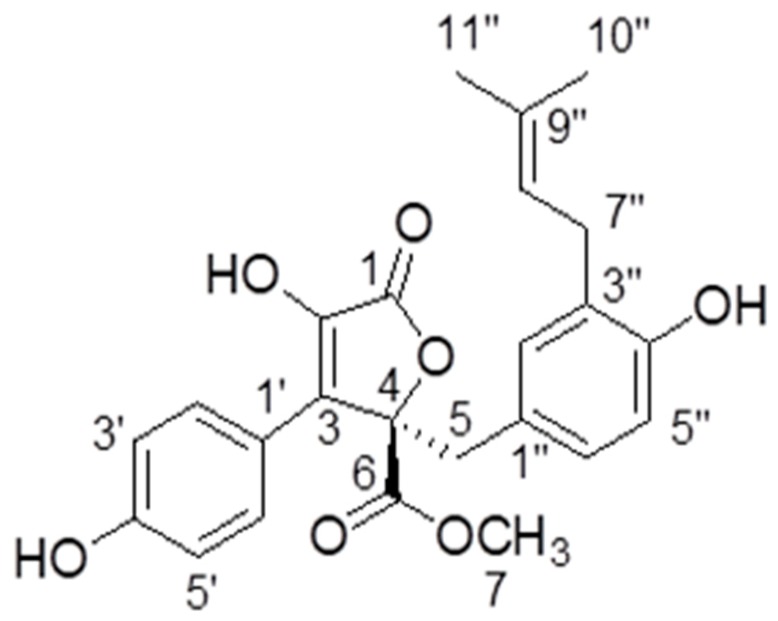
The structure of butyrolactone-I.

^1^H NMR (CD_3_OD, 700 MHz): δ_H_ 7.59 (H-2′ and H-6′, d, 8.4), 6.87 (H-3′ and H-5′,d, 9.1), 6.54 (H-6″, dd, 8.1, 2.1), 6.50 (H-5″, d, 8.4), 6.41 (H-2″, d, 2.1), 5.07 (H-8″, m), 3.78 (H-7, s), 3.43 (H-5, d, 14.0), 3.08 (H-7″, m), 1.67 (H-10″, s), 1.57 (H-11″, s). ^13^C-NMR NMR (CD_3_OD, 175 MHz): δ_C_ 171.41 (C-6), 170.11 (C-1), 159.13 (C-4′), 154.88 (C-4″), 139.46 (C-2), 132.78 (C-9″), 132.19 (C-2″), 130.19 (C-2′ and C-6′), 129.56 (C-3″), 129.01 (C-3), 128.23 (C-6″), 124.85 (C-1″), 123.36 (C-8″), 122.94 (C-1′), 116.41 (C-3′ and C-5′), 114.83 (C-5″), 86.59 (C-4), 53.64 (C-7), 39.41 (C-5), 28.48 (C-7″), 25.75 (C-10″), 17.56 (C-11″) (Figure 1). $[\alpha {]}_{D}^{20}$ + 108.85° (c 0.2, MeOH).

### 2.2. Effect of ZB5-1 on BV-2 Cell Viability

**Figure 2:**
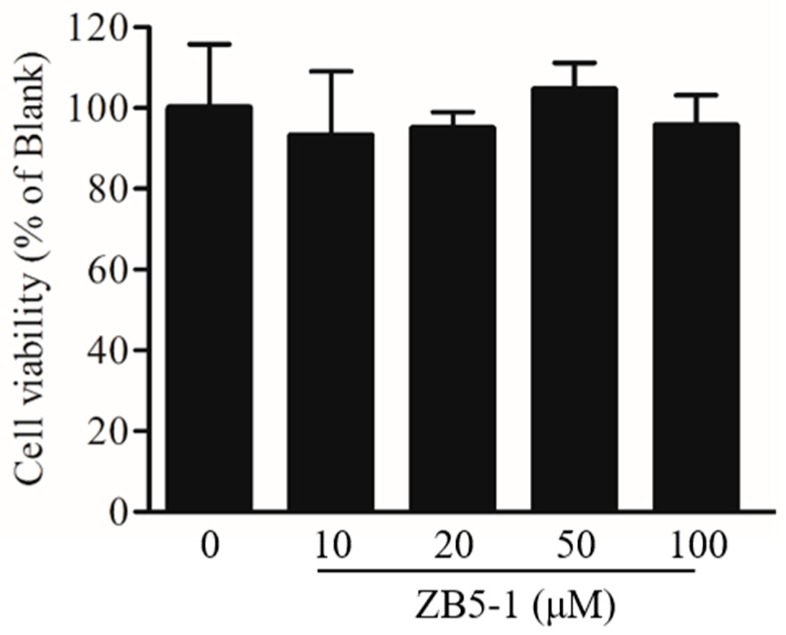
Effect of butyrolactone-I (ZB5-1) on the viability of BV-2 cells. The cells were treated with ZB5-1 (10, 20, 50, and 100 μM) for 24 h. Cell viability was measured using MTT assay.

The effect of ZB5-1 on BV-2 cell viability was measured by MTT assay. BV-2 cells were treated with different concentrations of ZB5-1 (10, 20, 50, and 100 μM). As shown in Figure 2, in the tested concentrations, ZB5-1 had no cytotoxicity on BV-2 cells. Therefore, concentrations of 10, 20, 50, and 100 μM of ZB5-1 were used for the remainder of the study.

### 2.3. Effect of ZB5-1 on NO Production in BV-2 Cells

**Figure 3:**
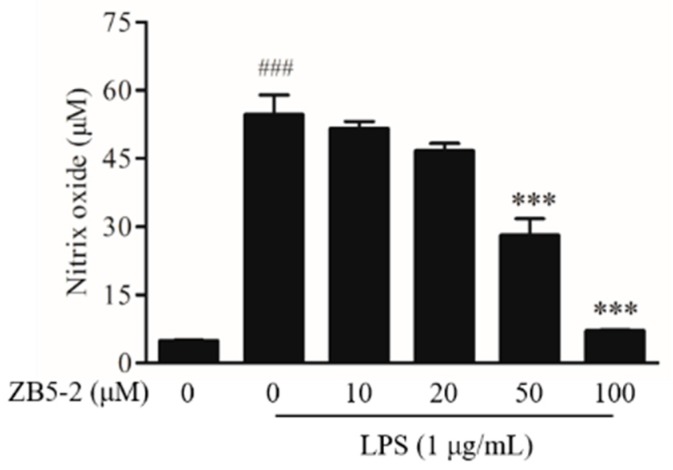
Inhibitory effect of ZB5-1 on nitric oxide (NO) production of BV-2 cells. The cells were pre-treated with various concentration of ZB5-1 (10, 20, 50, 100 μM) for 1 h, and then stimulated by lipopolysaccharide (LPS) (1 μg/mL) for another 24 h. The production of NO was measured using Griess reagent reaction. The results were expressed as the mean ± SD (*n* = 3). ### *p* < 0.001 compared with Blank; *** *p* < 0.001 compared with Control.

Griess reagent reaction was used to assess the level of NO of BV-2 cells treated with and without lipopolysaccharide (LPS) and ZB5-1. Figure 3 presented the experimental data of NO production. In resting state, BV-2 produced a small amount of NO. Once activated, the production of NO was reached at about 50 μM, a significant difference when compared with the resting state. When the cells were pre-treated with increasing concentrations of ZB5-1, the levels of NO were decreased. These results indicated that ZB5-1 could have a potential anti-inflammatory effect.

### 2.4. Effect of ZB5-1 on the Expression of iNOS and COX-2 in BV-2 Cells

**Figure 4:**
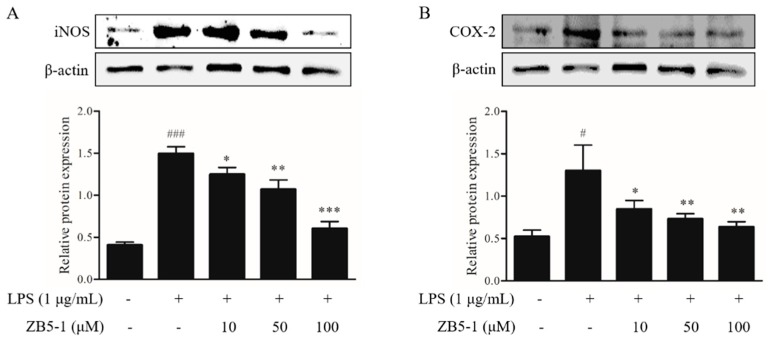
Inhibitory effect of ZB5-1 on protein expression of nitric oxide synthase (iNOS) (**A**) and cyclooxygenase-2 (COX-2) (**B**) of BV-2 cells. The cells were pre-treated with various concentration of ZB5-1 (10, 50, 100 μM) for 1 h, and then stimulated by LPS (1 μg/mL) for another 24 h. Expression of iNOS and COX-2 were determined by Western blot. The results were expressed as the mean ± SD (*n* = 3). ### *p* < 0.001, # *p* < 0.05 compared with Blank; *** *p* < 0.001, ** *p* < 0.01, * *p* < 0.05 compared with Control.

To investigate the inhibitory effect of ZB5-1 on the protein expression of inflammatory enzymes nitric oxide synthase (iNOS) and cyclooxygenase-2 (COX-2), the cells were pretreated with the indicated concentrations of ZB5-1 (10, 50, and 100 μM) before stimulated with LPS (1 μg/mL). As shown in Figure 4, ZB5-1 treatment decreased the levels of iNOS and COX-2. In particular, iNOS seemed to be more sensitive than COX-2 in BV-2 cells.

### 2.5. Effect of ZB5-1 on IL-1β Production in BV-2 Cells

**Figure 5:**
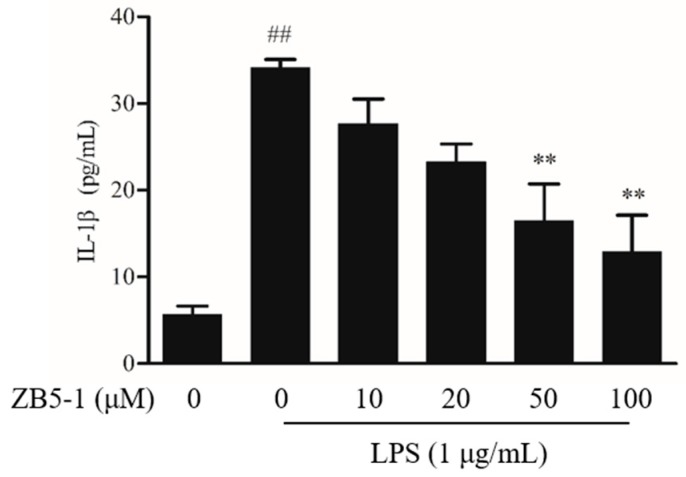
Inhibitory effect of ZB5-1 on interleukin-1 beta (IL-1β) level of BV-2 cells. The cells were pre-treated with various concentration of ZB5-1 (10, 20, 50, and 100 μM) for 1 h, and then stimulated by LPS (1 μg/mL) for another 24 h. The levels of IL-1β was determined by ELISA. The results were expressed as the mean ± SD (*n* = 3). ## *p* < 0.01 compared with Blank; ** *p* < 0.01 compared with Control.

To investigate the inhibitory effect of ZB5-1 on interleukin-1 beta (IL-1β), the cells were treated with LPS in the absence or presence of ZB5-1, and the levels were measured by enzyme-linked immunosorbent assay (ELISA). As shown in Figure 5, compared with unstimulated cells, the levels of IL-1β were increased in the culture media of LPS-induced cells, which were reduced by ZB5-1 in a dose-dependent manner.

### 2.6. Effect of ZB5-1 on NF-κB Phosphorylation of BV-2 Cells

**Figure 6:**
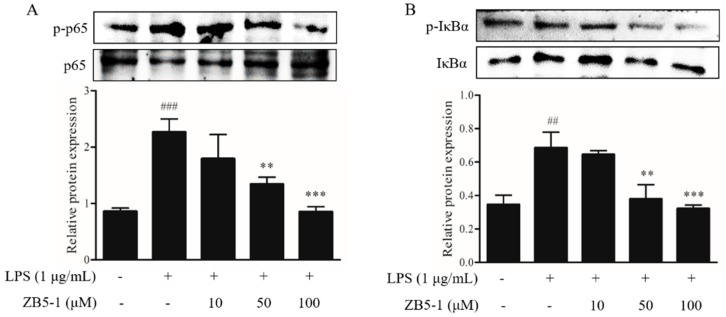
Inhibitory effect of ZB5-1 on the phosphorylation of NF-κB p65 (**A**) and IκBα (**B**) of BV-2 cells. The cells were pre-treated with various concentrations of ZB5-1 (10, 50, and 100 μM) for 1 h, and then stimulated by LPS (1 μg/mL) for another 24 h. The expression of p-IκBα and p-p65 were determined by Western blot analysis. The results were expressed as the mean ± SD (*n* = 3). ### *p* < 0.001, ## *p* < 0.01 compared with Blank; *** *p* < 0.001, ** *p* < 0.01 compared with Control.

To investigate whether ZB5-1 inhibits the nuclear factor-κB (NF-κB) signaling pathway, the phosphorylation of NF-κB p65 and IκBα were measured. As shown in Figure 6, phosphorylation of NF-κB p65 and IκBα were dramatically increased when the cells were exposed to the LPS, however, they were down-regulated when the cells were pre-treated with ZB5-1.

### 2.7. Effect of ZB5-1 on NF-κB Translocation in BV-2 Cells

**Figure 7:**
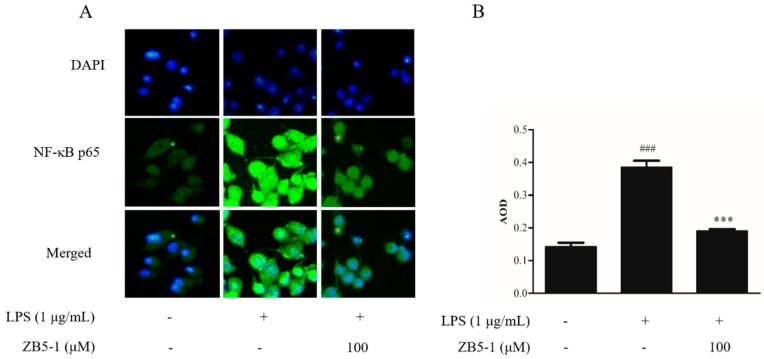
The effect of ZB5-1 on translocation of NF-κB p65 in BV-2 cells. The cells were pre-treated with 100 μM ZB5-1 for 1 h, and then stimulated by LPS (1 μg/mL) for another 24 h. Nuclear translocation of NF-κB p65 was monitored by an overlay of blue DAPI staining with green p65 immunofluorescence (**A**). The average optical density (AOD) of fluorescence was measured by Image J and the results were expressed as the mean ± SD (*n* = 3). ### *p* < 0.001, compared with Blank; *** *p* < 0.001, compared with Control (**B**).

The immunocytochemistry was availed to observe the location of a NF-κB p65 subunit in BV-2 cells. The nucleus was stained with DAPI, shown in Figure 7 in blue, while the p65 protein was incubated by an anti-NF-κB p65 primary antibody and stained by DyLight 488 AffiniPure goat anti-rabbit IgG secondary antibody, shown in Figure 7 in green. Figure 7 shows the location of the NF-κB p65 subunit. In the blank group, the p65 protein existed outside the nucleus with weak immunofluorescence. However, when the cells were exposed to LPS, the immunofluorescence became brighter than in the blank group and approached the nucleus, which meant LPS stimulation induced the translocation of p65 from cytoplasm to nucleus. The ZB5-1 exposure (100 μM) decreased the translocation.

### 2.8. Docking of ZB5-1 with NF-κB and COX-2

**Figure 8:**
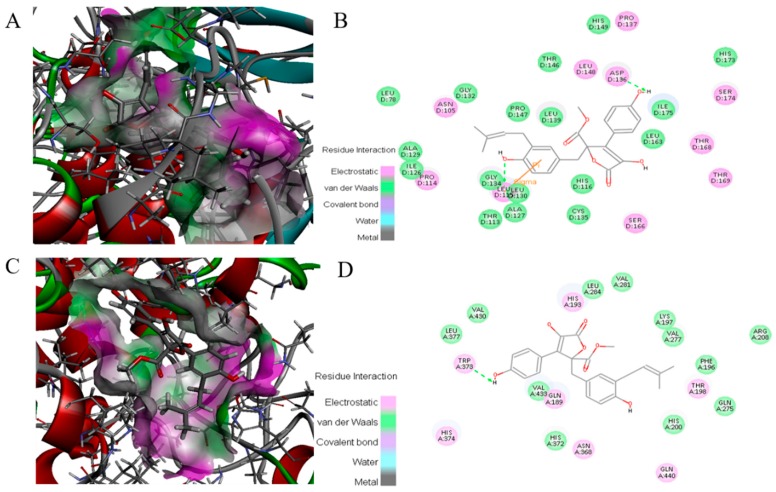
Binding mode of ZB5-1 into the NF-κB p65 (**A**) and 2-D depiction of the binding mode of ZB5-1 showing interactions formed with important residues. Hydrogen bonds formed with GLY 134 and ASP 136 (green arrow) (**B**). Binding mode of ZB5-1 into the COX-2 (**C**) and 2-D depiction of the binding mode of ZB5-1 showing interactions formed with important residues. Hydrogen bonds formed with TRP 373 (green arrow) (**D**).

To investigate the molecular mechanism for the binding of ZB5-1 and NF-κB along with COX-2, docking analysis of ZB5-1 within the NF-κB and COX-2 active site was performed using Discovery Studio (Figure 8). The obtained docking results showed that the docked pose of ZB5-1 with NF-κB has CDOCKER energy of −110.64 kcal/mol and CDOCKER interaction energy of −10.78 kcal/mol. ZB5-1 formed two hydrogen bonds with NF-κB. One of the hydrogen bonds is formed with ASP 136 with a 4.3 Å distance H-bond; the other hydrogen bond is formed with GLY 134 with a distance of 4.4 Å. In addition, the docked pose of ZB5-1 with NF-κB has CDOCKER energy of −0.38 kcal/mol and CDOCKER interaction energy of 43.40 kcal/mol, and a phenolic group formed one hydrogen bond with COX-2 at TRP 373 with a distance of 6.1 Å. Along with H-bonding, ZB5-1 established electrostatic interaction, i.e., van der Waals energies, with NF-κB and COX-2.

## 3. Discussion

The *Aspergillus* genus is a rich source of butyrolactone derivatives, which are formed by a 5-membered lactone ring and two benzene rings [22]. Butyrolactone I was firstly reported in 1977, isolated from *A. terreus* [23]. Previous studies indicated that butyrolactone I has various activities, including anti-proliferative activity against human lung and prostatic cancer cell lines. More importantly, it can inhibit cyclin-dependent kinases (CDKs), particularly CDK1 and CDK2, both of which participate in mammalian cell progression from G1 to S phase and from G2 phase to M phase respectively [24]. For example, butyrolactone I can induce neuronal differentiation by inhibiting the Neuro 2a cell cycle progression [25], and can also reduce the apoptosis of X-ray and Doxorubicin-induced DLD1 (p21+/+) human colorectal carcinoma cells [26]. In this study, we estimated the anti-neuroinflammatory effect on an LPS-stimulated BV-2 microglia cell.

Microglia cells can exert neuroprotective and neurotoxic functions in the brain. Activated by stimuli (e.g., LPS), they release pro-inflammatory factors such as NO, PGE_2_, chemokines, and cytokines [27], which cause neuronal death, and IL-6, which can up-regulate amyloid precursor protein synthesis and processing. These processes are considered hallmarks of neuro-inflammation involved in the pathological process of neurodegenerative diseases [28]. Therefore, reduction of microglial cell activation is conducive to prevent or treat neurodegenerative diseases [29].

NO is a short-life, ubiquitous molecular mediator, regulating a wide range of physiologic functions from neurotransmission, gastric motility, wound healing, mitochondrial respiration, apoptosis, and inflammation [30]. It has been well-documented that NO is produced from L-arginine catalyzed by a class of nitric oxide synthases (NOS), namely neuronal NOS (nNOS or NOS1), endothelial NOS (eNOS or NOS3), and inducible NOS (iNOS or NOS2) [31]. According to its origin and production, NO exerts bifacial influences on physiological processes. Low amounts of NO derived from eNOS and nNOS is beneficial, whereas prodigious amounts of NO synthesized from iNOS are observed in different experimental models of inflammation [32,33]. Several studies suggested that over-activated microglia cells can release over-production of NO, which can induce neurotoxic injury to neurons and contribute to neurodegenerative diseases [3,34]. In this study, ZB5-1 had the ability to down-regulate the production of NO (Figure 3). Furthermore, it also inhibited LPS-induced iNOS protein levels in a dose-dependent manner (Figure 4A). IL-1 is one of the major pro-inflammatory cytokines produced by activated BV-2 cells, and its over-production has a relationship with neuronal cell damage and many neurodegenerative diseases. Thus, inhibition of IL-1β production serves as a key mechanism in the control of CNS inflammation [35]. This study showed that ZB5-1 inhibited the secretion of IL-1β (Figure 5). Furthermore, we also investigated the inhibitory effect of ZB5-1 on the expression of COX-2, a key enzyme responsible for the conversion of arachidonic acid into prostaglandin E_2_ (PGE_2_) [36]. Previous studies showed that the expression of COX-2 was elevated during inflammation in neurons and activated microglia cells in brain diseases, such as Alzheimer’s disease (AD) and Parkinson’s disease (PD) [37,38]. Therefore, COX-2 has been deemed to be a target for anti-inflammatory drugs. Several epidemiological studies indicated that treatment with NSAIDs (nonsteroidal anti-inflammatory drugs) can reduce the risk of AD because NSAIDs can delay its onset or slow its progression [39,40]. In addition, treatment with COX-2 inhibitors could reduce the inflammatory reaction and increase survival of neurons, decreasing the level of PGE_2_ [41].

NF-κB plays a crucial role in host immunity and is a central regulator of microglial responses to stimuli, including LPS and cytokines [42], and its activation in microglia contributes to neuronal injury and promotes the development of neurodegenerative disorders. In addition, ZB5-1 was able to inhibit the phosphorylation of p65, the degradation of IκBα, and the nuclear translocation of p65 in LPS-induced BV-2 cells.

Docking is a powerful and theoretical method for the discovery of interactions between molecules and proteins [43]. In our docking studies, a total of 10 random conformations were generated for ZB5-1 in the receptor cavity through high temperature molecular dynamics, preceded by random rotations. In the best molecular interaction pose of ZB5-1 and NF-κB (CDOCKER energy of −110.64 kcal/mol and CDOCKER interaction energy of −10.78 kcal/mol), two hydrogen bonds were formed with ASP136 and GLY134, both of which are amino acid residues of NF-κB. The CDOCKER energy was with high negative values, indicating strong interactions between ZB5-1 and NF-κB [44]. The energy profile suggests that ZB5-1 formed strong interactions with active site residues of NF-κB due to hydrogen bonds. This study clearly indicates that ZB5-1 might be a good inhibitor of NF-κB that will not degrade by exposure to LPS stimulation.

Regarding the compound structure, ZB5-1 has a furanone ring system. Also known as butyrolactone or butanolide, ZB5-1 is a widely recognized component of natural products, exhibiting an extensive spectrum of pharmacological activities [45]. Moreover, a quantitative structure-activity relationship (QSAR) analysis assessed that furanone derivatives would be a potential inhibitor of COX-2 [46]. There are two phenolic groups in ZB5-1, and it has been proved that phenolic compounds possess good anti-inflammatory effects [47]. In the case of ZB5-1 with COX-2, we observed that its CDOCKER energy is −0.38 kcal/mol, however, the CDOCKER interaction energy is 43.40 kcal/mol, and it forms only one hydrogen bond with TRP373 of COX-2. Due to its small molecule size, it could be suggested that the compound bioavailability would be high, and that it would have the possibility of passing the blood brain barrier (BBB) to reach microglia cells in the brain [48].

## 4. Materials and Methods

### 4.1. Reagents

Lipopolysaccharide (LPS), 3-(4, 5-dimethylthiazol-2-yl)-2, 5-diphenyl-2Htetrazolium bromide (MTT), and dimethylsulfoxide (DMSO) were purchased from Sigma-Aldrich (St. Louis, MO, USA). The Griess Reagent System was obtained from Promega Corporation (Madison, WI, USA). The commercial-specific complete Dulbecco’s Modified Eagle’s Medium (DMEM), penicillin, and streptomycin were purchased from GIBCO (Carlsbad, CA, USA). The ECL kit was provided from GE Healthcare. 4′,6-diamidino-2-phenylindole (DAPI) was obtained from Solarbio (Beijing, China); primary and secondary antibodies used for western blot were purchased from Santa Cruz Biotechnology Inc (Santa Cruz, CA, USA). The anti-NF-κB p65 antibody was obtain from Proteintech Group (Rosemont, IL, USA); Dylight 488 conjugated Goat Anti-Rabbit IgG secondary antibody was purchased from Abbkine (Redlands, CA, USA). The commercial ELISA kit for detecting IL-1β was obtained from Beyotime Biotechnology (Nanjing, China). Those chemicals not mentioned in present research were of analytic grade.

### 4.2. Strain, Fermentation, and Purification

The producer strain XWC21-10 originated from coral *Porites pukoensis* in Zhanjiang seawaters of the South China Sea, identified as *Aspergillus terreus* by ITS rDNA sequencing with Genbank number JQ717327, and deposited in the Guangdong culture collection center with accession number GDMCC No. 60004.

This strain was fermented in 1L flasks with the total scale of 40 L in potato dextrose broth (PDB) medium at 28 °C and 150 rpm on a shaker for a week. After fermentation, the culture was filtrated to separate broth and mycelia, which were respectively extracted by ethyl acetate and ethyl acetate plus chloroform-methanol (1:1, *v*/*v*) mixture. The two extracts were finally combined and condensed in vacuo to a crude extract (18.7 g). This extract was separated on a Sephadex LH-20 column eluted by chloroform-methanol (1:1, *v*/*v*) to provide 9 fractions (Fr 1 to Fr 9). The Fr 7 was further purified by a Sephadex LH-20 column eluted by methanol and then by preparative RP-18 HPLC eluted with 70:30 methanol–water to yield pure compound ZB 5-1 (17.8 mg).

### 4.3. Cell Culture and Cell Viability Assay

The mouse BV-2 microglia cell were obtained from the Cell Bank of Chinese Academy of Sciences (Shanghai, China). The cells were cultured in DMEM supplemented with 5% FBS and 1% of penicillin/streptomycin at 37 °C in a humidified 5% CO_2_ incubator. The cytotoxicity of ZB5-1 on BV-2 cells were measured by MTT assay as previously described [49]. Briefly, BV-2 cells were seeded at density 1 × 10^4^ cells/mL in 96-well plates and were treated with ZB5-1 (10, 20, 50, 100 μM) for 24 h. Then 100 μL MTT (1 mg/mL) was added into the cell culture. Four hours later, the formazan crystals were dissolved in DMSO and the absorbance at 540 nm were investigated using a microplate reader (BioTek, Winooski, VT, USA). The results were expressed as a percentage of surviving cells over control cells.

### 4.4. Nitric Oxide Determination

To examine the inhibitory effect of ZB5-1 on the production of NO, BV-2 cells were seeded at density 5 × 10^4^ cells/well in 96-well plates. The cells were pre-treated with ZB5-1 (10, 20, 50, and 100 μM) for 1 h. They were then activated by LPS (1 μg/mL) for 24 h. The productions of NO were measured using the Griess Reagent System according to previous study [50] protocol.

### 4.5. Cytokine Assay

The level of IL-1βwas determined by ELISA as previously described [51]. In brief, BV-2 cells were seeded at 5 × 105 cells/mL in 24-well plates for 24 h. The cells were pre-treated with ZB5-1 (10, 20, 50, and 100 μM) for 1 h and then activated by LPS (1 μg/mL) for another 24 h. The culture medium was collected and centrifuged at 12,000 rpm for 10 min. The level of IL-1β in the culture medium was quantified using ELISA kits (Beyotime Biotechnology, Shanghai, China).

### 4.6. Western Blot Analysis

Whole cell protein extraction and western blotting were performed as previously described [44]. BV-2 cells were seeded at 5 × 10^6^ cells/mL in 6-well plates for 24 h. The cells were pre-treated with ZB5-1 (10, 50, and 100 μM) for 1 h and then activated by LPS (1 μg/mL) for another 24 h. The collected cells were rinsed three times with cold phosphate buffer saline (PBS), and total cell lysates were obtained by adding 100 μL of RIPA buffer. Protein concentrations were determined using a Pierce BCA Protein Assay Kit (Thermo Fisher Scientific). Equal amounts of protein (20–40 µg) were separated electrophoretically using a 10% sodium dodecyl sulfate-polyacrylamide gel electrophoresis (SDS-PAGE), and the proteins were transferred onto nitrocellulose (NC) membranes (Amersham). Membranes were then blocked with 5% skim milk and incubated with the primary antibody and the horseradish peroxidase (HRP)-conjugated secondary antibody. Blots were quantified by an Amersham ECL Western Blotting Detection Kit (GE Healthcare).

### 4.7. Immunocytochemistry

Immunocytochemistry was employed to investigate the translation of NF-κB p65 [51]. BV-2 cells were seeded at 5 × 10^4^ cells/mL in 24-well plates for 24 h. The cells were pre-treated with ZB5-1 (100 μM) for 1 h and then activated by LPS (1 μg/mL). After a 24 h exposure period, cells were washed three times with cold PBS (0.1 M) and were fixed for 10 min in 4 °C with ice cold 4% paraformaldehyde (PFD). Cells were then washed with PBS (0.1 M) and permeabilized with 0.2% Triton X-100 for 10 min in 4 °C. The cells were blocked with 5% bovine serum albumin (BSA) for 1 h at room temperature (RT). The plates were then incubated overnight at 4 °C with anti-NF-κB p65 (rabbit monoclonal IgG, 1:100 dilution). After removing the primary antibody, the cells were washed again and incubated with Dylight 488 conjugated Goat Anti-Rabbit IgG secondary antibody (1:500 dilution) for 2 h at room temperature (25 °C). Finally, cells were counterstained for 5 min with 5.0 μg/mL DAPI and washed with PBS. Images were taken using an inverted fluorescence microscope with the imaging software Cell Sens (Olympus Opticals, Tokyo, Japan).

### 4.8. Molecular Docking

Molecular docking between ZB5-1 and NF-κB along with COX-2 was performed by Discovery Studio 3.5 (Accelrys, San Diego, CA, USA). The crystal structures of NF-κB (PDB ID: 1IKN) and COX-2 (3LN1) were obtained from the PDB database. The structure of ZB5-1 was drawn with Chemdraw. The protein and ligand molecules were prepared by DS 3.5 before docking. Molecular docking of the ZB5-1 into the NF-κB and COX-2 proteins binding sites were performed using CDOCKER protocol. The negative CDOCKER energy was calculated after conducting the molecular docking [52].

### 4.9. Statistics

For the statistical analyses, values are expressed as the means ± SD (*n* = 3). The statistical significance was determined by one-way ANOVA and *t*-test. A value of *p* < 0.05 was considered statistically significant.

## 5. Conclusions

In conclusion, our results reveal that ZB5-1 has an anti-neuroinflammatory effect on LPS-induced BV-2 cells. Our results indicated that ZB5-1 significantly reduced LPS-induced NO production in BV2 microglia cells and also inhibited the expression of iNOS and COX-2. Additionally, ZB5-1 suppressed LPS-induced phosphorylation and nuclear translocation of NF-κB in BV2 microglia cells. Docking study showed that molecular events occurred at the binding interface of ZB5-1 with NF-κB and COX-2 interaction sites. Therefore, our results suggest that ZB5-1 may act as a potential candidate for treating inflammation-related neurological disorders and provide a new insight into the secondary metabolism derived from marine fungi.
